# *RNF213* p.Arg4810Lys Variant Is Associated with Higher Stenosis Progression in Asymptomatic Intracranial Artery Stenosis

**DOI:** 10.1007/s12975-024-01309-x

**Published:** 2024-11-12

**Authors:** Shogo Dofuku, Satoru Miyawaki, Hideaki Imai, Masahiro Shimizu, Hiroki Hongo, Yuki Shinya, Kenta Ohara, Yu Teranishi, Hideaki Ono, Hirofumi Nakatomi, Akira Teraoka, Nobuhito Saito

**Affiliations:** 1 Department of Neurosurgery, Faculty of Medicine, The University of Tokyo, Tokyo, Japan; 2 Department of Neurosurgery, Tokyo Shinjuku Medical Center, Tokyo, Japan; 3 Department of Neurosurgery, Kanto Neurosurgical Hospital, Kumagaya, Japan; 4 Department of Neurologic Surgery, Mayo Clinic, Rochester, MN USA; 5 Department of Neurosurgery, Fuji Brain Institute and Hospital, Fujinomiya, Japan; 6 Department of Neurosurgery, Faculty of Medicine, The University of Kyorin, Tokyo, Japan; 7 Department of Neurosurgery, Teraoka Memorial Hospital, Fukuyama, Japan

**Keywords:** Ischemic stroke, Stenosis, *RNF213*

## Abstract

Intracranial artery stenosis (ICAS) is a significant contributor to ischemic stroke, with the *RNF213* p.Arg4810Lys variant identified as a related genetic factor. We explored the clinical outcomes of the *RNF213* genotype in patients with asymptomatic ICAS. Between November 2011 and March 2019, 139 patients with asymptomatic ICAS were enrolled in this study. Genotyping for *RNF213* p.Arg4810Lys was performed using Sanger sequencing. A comprehensive analysis was conducted to compare the *RNF213* genotype with background characteristics and clinical outcomes such as ipsilateral ischemic cerebrovascular events and stenosis progression. *RNF213* p.Arg4810Lys was found in 25% of cases, revealing distinct clinical features between carriers and non-carriers. The incidence of ipsilateral ischemic cerebrovascular events was 4.3% (6/139 cases), and stenosis progression was observed in 13% (18/139 cases) during a mean follow-up period of 58 months. Stenosis progression rates were notably higher in the *RNF213* variant group (25.7%; 9/35 cases) than in the *RNF213* wild-type group (8.7%; 9/104 cases). Cumulative stenosis progression rate was significantly higher in the *RNF213* variant group than in the *RNF213* wild-type group (log-rank test, *P* = 0.0004). Multivariate Cox regression analysis indicated a significant association between the *RNF213* p.Arg4810Lys variant and an increased risk of stenosis progression (*P* = 0.03, odds ratio 3.2; 95% confidence interval, 1.1–9.0). The *RNF213* p.Arg4810Lys variant exhibits clinical disparities in asymptomatic ICAS and is notably linked to a heightened risk of stenosis progression. These results suggest a distinct difference in the vascular stenosis mechanism associated with this variant, warranting further investigation into its clinical implications and potential mechanistic insights.

## Introduction

Intracranial artery stenosis (ICAS) is a leading global cause of ischemic stroke [1]. Atherosclerotic changes due to lifestyle-related diseases contribute to ICAS development [2]. Factors like age, hypertension, diabetes mellitus, and dyslipidemia are correlated with ICAS prevalence, especially in regions like East and South Asia, where it comprises up to half of all ischemic stroke cases [3, 4]. Regarding genetic background, multiple single variant association studies have identified the *RNF213* p.Arg4810Lys variant (rs112735431, c.14429G > A) as the most influential genetic factor linked to ICAS [5, 6]. Notably, clinical features of patients with ICAS have been reported to vary based on the *RNF213* p.Arg4810Lys variant [7]. Moreover, a genome-wide association study of ICAS highlighted that the *RNF213* p.Arg4810Lys variant is the most significant variant associated with ICAS [8].

Aggressive medical treatment and management of the modifiable risk factors to prevent recurrence are crucial in addressing symptomatic ICAS, which leads to transient ischemic attacks and cerebral infarctions [9]. In contrast, asymptomatic ICAS has become more prominent due to widespread magnetic resonance imaging (MRI) usage [10]. However, despite increased awareness, comprehensive data on the prognosis and optimal treatment for asymptomatic ICAS are still limited [11]. Understanding the link between the genetic variant and clinical outcomes is vital for refining treatment strategies for patients with asymptomatic ICAS. Therefore, this study aimed to examine the association between the *RNF213* p.Arg4810Lys variant and long-term clinical outcomes in patients with asymptomatic ICAS, focusing on the progression of ipsilateral ischemic stroke and stenosis during the follow-up period.

## Materials and Methods

### Study Population

We retrospectively collected data from 408 patients diagnosed with ICAS between November 2011 and March 2019 at The University of Tokyo, Kanto Neurosurgical Hospital in Saitama, and Teraoka Memorial Hospital in Hiroshima. The criteria for ICAS were as follows: (i) stenosis or occlusion in major intracranial arteries on imaging findings (detailed below); (ii) age > 40 years; (iii) one or more risk factors for atherosclerosis, such as hypertension, diabetes mellitus, dyslipidemia, coronary artery disease, arteriosclerosis obliterans, or a history of smoking; and (iv) no signs of cardiac embolism, dissection, vasculitis, or moyamoya disease (MMD) or quasi-MMD.

**Fig. 1:**
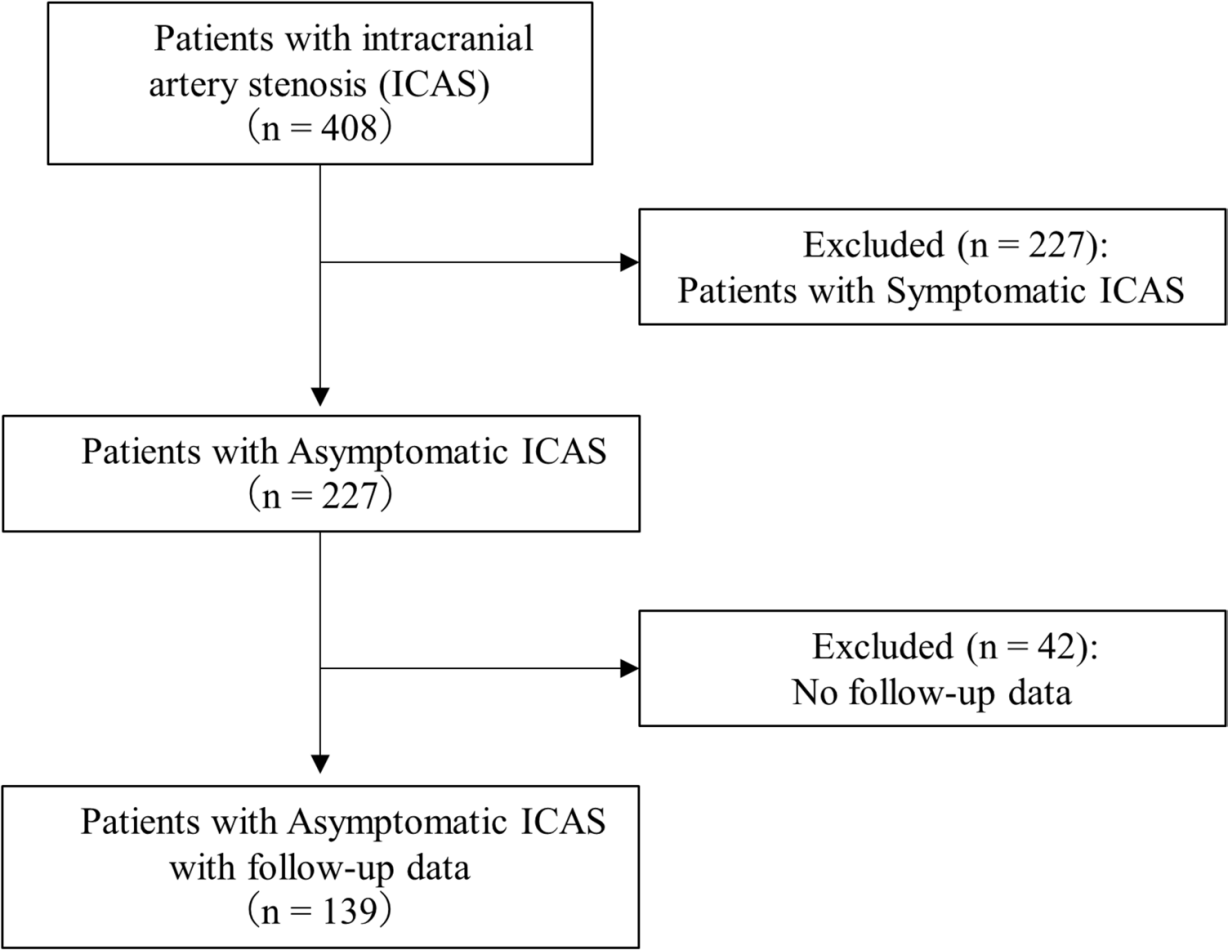
Flow diagram of patient selection

Among these, we excluded patients with symptomatic ICAS diagnosed with cerebral infarction or transient ischemic attack in the stenotic artery area. Conversely, patients with asymptomatic ICAS were defined as those who underwent a medical checkup or exhibited unrelated symptoms such as headache and dizziness. We also excluded patients without follow-up MRA. Figure 1 shows a flow diagram of patient selection. Data on age, sex, hypertension, diabetes mellitus, dyslipidemia, coronary artery disease, arteriosclerosis obliterans, alcohol and smoking history, and family history of stroke were collected from the medical records. Hypertension was defined as systolic blood pressure ≥ 140 mmHg, diastolic blood pressure ≥ 90 mmHg, or the use of antihypertensive agents. Diabetes mellitus was defined as a HbA1c level ≥ 6.5% or taking anti-diabetic medications. Dyslipidemia was defined as either a high-density lipoprotein cholesterol level < 40 mg/dL, a low-density lipoprotein cholesterol level ≥ 140 mg/dL, a triglyceride level ≥ 150 mg/dL, or receiving lipid-lowering treatment.

### Imaging Findings

ICAS diagnosis was primarily based on magnetic resonance angiography (MRA) (1.5 T or 3 T) or digital subtraction angiography (DSA). Two or more physicians, including at least one neurosurgeon and radiologist, reviewed the MRA images. The study excluded patients diagnosed with non-atherosclerotic ICAS such as dissection, arteritis, and MMD. Additionally, we collected information on the stenosis degree and site and ipsilateral ischemic cerebrovascular event (cerebral infarction or transient ischemic attack) onset during the follow-up period from the medical records and MRI or DSA. The stenosis site was classified as the internal carotid artery, middle cerebral artery, basilar artery, vertebral artery, and others. The horizontal segment was defined as M1 stenosis, particularly in the middle cerebral artery. Proximal stenosis was defined as internal carotid artery and M1 stenosis, and multiple lesions were defined as stenosis in two or more locations. The stenosis degree in each patient on MRA or DSA was calculated using the criteria established in the WASID Trial and the TOSS Trial [12, 13]. The extent of stenosis in each patient was classified into five grades by consensus: normal, mild (signal reduction < 50%), moderate (signal reduction ≥ 50%), severe (focal signal loss with the presence of distal artery signal), and occlusion [13]. Stenosis progression was defined as stenosis worsening by one or more grades on follow-up MRA as compared with the baseline MRA using the criteria established in the TOSS Trial [13]. The analysis excluded cases with occlusion at the time of diagnosis.

### Genetic Analysis

Peripheral blood samples were obtained from all enrolled patients. Genomic DNA was extracted from peripheral blood leukocytes at SRL, Inc. (Tachikawa, Tokyo, Japan), using a DNA Extraction Kit (Talent Srl, Trieste, Italy). The *RNF213* p.Arg4810Lys variant (rs112735431, c.14429G > A) (GenBank accession number, NM_020914.4) was screened by direct sequencing at FASMAC Co, Ltd. (Atsugi, Kanagawa, Japan) using an ABI Genetic Analyzer 3130XL or ABI DNA Analyzer 3730xL (Applied Biosystems, Foster City, CA) and analyzed using Sequence Scanner version 1.0 (Applied Biosystems). The same primer sequences and polymerase chain reaction conditions were used as previously described [14]. The investigators involved in genotyping were blinded to the patient’s information. All sequencing data analyses were performed at the Department of Neurosurgery at The University of Tokyo.

### Statistical Analysis

We compared the clinical features of the two groups: carriers of the *RNF213* p.Arg4810Lys variant (*RNF213* variant group) and non-carriers (*RNF213* wild-type group), based on the presence or absence of the risk allele of the *RNF213* p.Arg4810Lys variant using Fisher’s exact test. Mean age was compared using the Wilcoxon rank-sum test. The cumulative incidence rate of ipsilateral ischemic cerebrovascular events and stenosis progression during the follow-up period was assessed and stratified using the Kaplan–Meier method, and survival curves were compared using the log-rank test. We used the Cox proportional hazards model to analyze the risk factors for ipsilateral ischemic cerebrovascular events and stenosis progression using univariate and multivariable analyses. Variables with *P* ≤ 0.1 in the univariate analysis were chosen for inclusion in the multivariable model. If none of the variables met this criterion, we deliberately selected variables based on the previous literature and included age and sex in the model. Statistical significance was set at *P* < 0.05. Analyses were performed using JMP® 14 software (SAS Institute Inc., Cary, NC).

## Results

**Fig. 2:**
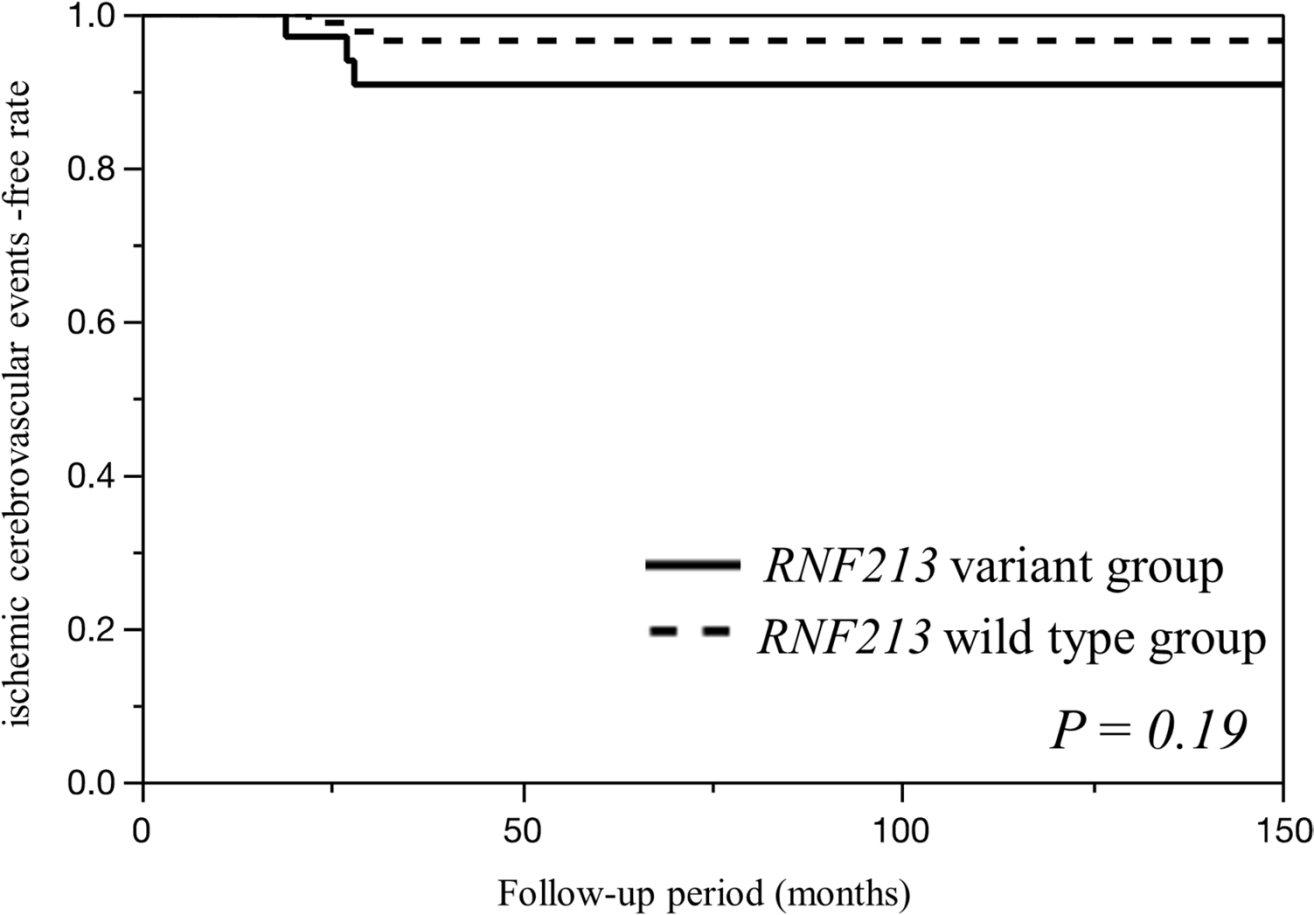
Kaplan–Meier curve for ischemic cerebrovascular event onset between the two groups with and without the risk allele of the *RNF213* variant

A total of 139 patients with asymptomatic ICAS were detected among 408 patients with ICAS. The *RNF213* p.Arg4810Lys variant (rs112735431, c.14429G > A) was detected in 25% of the individuals (35/139; 35 heterozygotes) who were significantly younger, had a family history of stroke, and had less diabetes mellitus than those without the risk allele (Table 1). The average follow-up period for ipsilateral ischemic cerebrovascular events was 58 months and the crude incidence rate was 4.3% (6/139 cases). Ipsilateral ischemic cerebrovascular events occurred in 8.6% (3/35) of the *RNF213* variant group and 2.9% (3/104 cases) of the *RNF213* wild-type group. The difference in the occurrence of ipsilateral ischemic cerebrovascular events with the variant in asymptomatic ICAS was statistically insignificant (log-rank test, *P* = 0.19), although the *RNF213* variant group exhibited a higher occurrence of ipsilateral ischemic cerebrovascular events than the *RNF213* wild-type group (Fig. 2).

**Table 1:** Clinical characteristics of the patients

	All (*n* = 139)	*RNF213* variant (*n* = 35)	*RNF213* wild-type (*n* = 104)	*P* value	Odds ratio (95% CI)
Mean age	63.3 ± 11.8	59.2 ± 10.5	64.7 ± 11.9	0.02^†^	
Age > 65 years	70 (50%)	10 (29%)	60 (58%)	0.003	0.29 (0.13–0.67)
Male	70 (50%)	13 (37%)	57 (55%)	0.08	0.49 (0.33–1.07)
Hypertension	96 (69%)	22 (63%)	74 (71%)	0.40	0.69 (0.31–1.54)
Diabetes mellitus	37 (27%)	3 (9%)	34 (33%)	0.004	0.19 (0.06–0.68)
Dyslipidemia	70 (50%)	16 (46%)	54 (52%)	0.56	0.78 (0.36–1.68)
Coronary artery disease	17 (12%)	5 (14%)	12 (12%)	0.77	1.28 (0.42–3.92)
Smoking history	36 (26%)	8 (23%)	28 (27%)	0.66	0.79 (0.32–1.95)
Family history of stroke	42 (34%)	18 (62%)	24 (25%)	0.0006^†^	4.84 (2.01–11.7)
Location of stenosis				0.15	
Middle cerebral artery	85 (61%)	25 (71%)	60 (58%)		
Internal carotid artery	39 (28%)	8 (23%)	31 (30%)		
Vertebral artery	12 (9%)	1 (3%)	11 (11%)		
Others	3 (2%)	1 (3%)	2 (2%)		
Proximal stenosis	73 (53%)	21 (60%)	52 (50%)	0.30	1.5 (0.69–3.27)
Multiple lesions	78 (56%)	19 (54%)	59 (57%)	0.84	0.91 (0.42–1.96)
Anterior ICAS	125 (90%)	34 (97%)	91 (88%)	0.19	4.86 (0.61–38.6)
Bilateral stenosis	55 (40%)	12 (34%)	43 (41%)	0.55	0.74 (0.33–1.65)
PCA involvement cases	21 (15%)	7 (20%)	14 (13%)	0.41	1.61 (0.59–4.38)
M1 stenosis	63 (45%)	22 (63%)	41 (39%)	0.02	2.60 (1.18–5.73)
Severity of stenosis				0.44	
Mild	7 (5%)	2 (6%)	5 (5%)		
Moderate	57 (41%)	12 (34%)	45 (43%)		
Severe	46 (33%)	10 (29%)	36 (35%)		
Occlusion	29 (21%)	11 (31%)	18 (17%)		
Stenosis progression	18 (13%)	9 (26%)	9 (9%)	0.01^†^	3.65 (1.32–10.1)

^†^Statistical significance: *P* < 0.05, Fisher’s exact test, Wilcoxon rank-sum test

*CI* confidence interval, *PCA* posterior cerebral artery, *ICAS* intracranial arterial stenosis

**Fig. 3:**
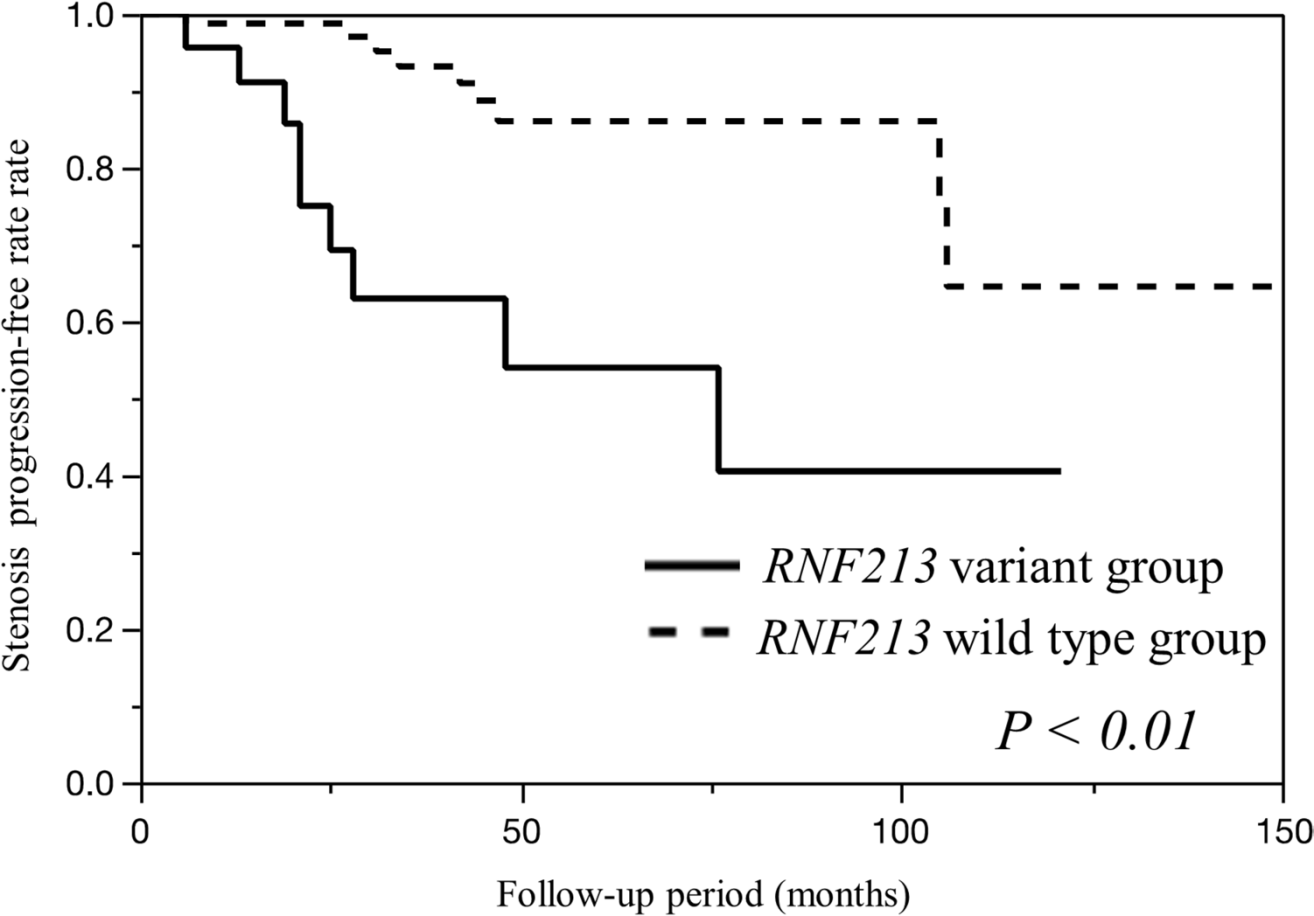
Kaplan–Meier curve for stenosis progression between the two groups with and without the risk allele of the *RNF213* variant

Eighteen (13%) patients developed stenosis. Stenosis progression occurred in 25.7% (9/35) of the *RNF213* variant group and 8.7% (9/104 cases) of the *RNF213* wild-type group. The difference in stenosis progression with the variant in asymptomatic ICAS was statistically significant (log-rank test, *P* = 0.0004) (Fig. 3). Univariate Cox regression analysis revealed that *RNF213* p.Arg4810Lys variant, age, and M1 stenosis were associated with a high risk of stenosis progression. Multivariable Cox regression analysis demonstrated that the *RNF213* p.Arg4810Lys variant (*P* = 0.03; odds ratio (OR), 3.2; 95% CI, 1.1–9.0) and M1 stenosis (*P* = 0.01; OR, 4.2; 95% CI, 1.3–13.3) were associated with a high risk of stenosis progression (Table 2).

**Table 2:** Risk factors of stenosis progression in ICAS patients

	Univariate analysis		Multivariable analysis	
	*P* value	Hazard ratio (95% CI)	*P* value	Hazard ratio (95% CI)
Age > 65 years	0.004^†^	0.16 (0.04–0.56)	0.18	0.39 (0.09–1.56)
Female	0.20	1.85 (0.71–4.80)	0.35	1.58 (0.59–4.24)
*RNF213* variant	0.001^†^	4.64 (1.82–11.7)	0.03^†^	3.16 (1.11–8.99)
M1 stenosis	0.002^†^	5.19 (1.82–14.7)	0.01^†^	4.22 (1.34–13.2)

^†^Statistical significance: *P* < 0.05, Cox proportional hazards model

*CI* confidence interval

## Discussion

Herein, we analyzed the association between the *RNF213* p.Arg4810Lys variant and the clinical characteristics and outcomes in patients with asymptomatic ICAS, focusing on the incidence of ipsilateral ischemic cerebrovascular events and stenosis progression. The longitudinal evaluation in this study, conducted over a mean follow-up period of 58 months, provided valuable insight into the clinical outcomes of asymptomatic ICAS. Among patients with asymptomatic ICAS, the ipsilateral ischemic cerebrovascular event incidence and stenosis progression were 4.3% (6/139 cases) and 13% (18/139 cases), respectively, over a mean follow-up period of 58 months. In addition, this analysis revealed distinct clinical features associated with the *RNF213* p.Arg4810Lys variant. The *RNF213* p.Arg4810Lys variant was associated with a high risk of stenosis progression particularly in the asymptomatic ICAS group, emphasizing its role in the vascular stenosis mechanism.

Asymptomatic ICAS is increasingly recognized due to the widespread use of MRI [10]. Furthermore, ICAS is associated with an increased risk of stroke and dementia [15]. Although the prevalence of asymptomatic ICAS is known to increase with age, data on the prognosis and optimal treatment for asymptomatic ICAS are limited [11]. Additionally, the clinical features of patients with symptomatic ICAS differ with and without the risk allele of the *RNF213* p.Arg4810Lys variant [7]. In this analysis, we focused on asymptomatic ICAS and analyzed the association between the *RNF213* p.Arg4810Lys variant and clinical characteristics and outcomes in patients with asymptomatic ICAS.

*RNF213* is a susceptibility gene for MMD in East Asian populations, characterized by idiopathic abnormal progressive stenosis of the intracranial arteries [14–17]. Moreover, the variant is associated with ICAS [5, 6]. The variant was previously identified in 17% of Japanese patients, 18% of Korean patients, and 2% of Chinese patients [18]. Several reports have indicated a correlation between the *RNF213* p.Arg4810Lys variant and clinical outcomes in patients with MMD [19–21]. Meanwhile, studies on the relationship between the *RNF213* p.Arg4810Lys variant and clinical outcomes in patients with ICAS remain limited [22, 23]. In this study, the *RNF213* variant group was substantially younger, had a family history of stroke, and less diabetes mellitus than the *RNF213* wild-type group. The vascular stenosis mechanism was assumed to differ between the groups. In addition, the *RNF213* p.Arg4810Lys variant was associated with a higher risk of stenosis progression in the asymptomatic ICAS group. Therefore, understanding the relationship between the *RNF213* p.Arg4810Lys variant and clinical outcomes may be critical for optimizing the treatment. Furthermore, gene searches may be useful as a treatment strategy for ICAS. The *RNF213* variant group is more likely to develop stenosis progression and requires closer follow-up.

On the other hand, it is interesting that the incidence rate of ischemic cerebrovascular event is similar between the two groups, despite more stenosis progression in the *RNF213* variant group. Although the *RNF213* variant group is more likely to develop stenosis of large arteries, it may be speculated that compensatory mechanisms, such as the development of collateral blood flow, play a role in mitigating the risk of ischemic stroke. Further investigation into the development and function of collateral circulation in the *RNF213* variant group could provide valuable insights into the pathophysiology of ischemic cerebrovascular events in this population.

The histology of ICAS and other systemic arteries is characterized by a lipid-rich plaque burden, intraplaque hemorrhage, fibrin cap, and inflammatory cell infiltration [24–27]. ICAS associated with the *RNF213* variant has been reported to show negative remodeling [28, 29]. However, detailed histological findings of wall shrinkage in the negative remodeling of ICAS with the *RNF213* variant have not been elucidated. Further histological observations are required to investigate the effects of *RNF213* p. Arg4810Lys on ICAS in detail. Moreover, functional analysis of the RNF213 protein remains a critical issue*.*

Recent reports have linked the *RNF213* p.Arg4810Lys variant to coronary artery stenosis, pulmonary artery stenosis, pulmonary arterial hypertension, and renal artery stenosis [30–33]. In particular, the *RNF213* p.Arg4810Lys variant was associated with adverse clinical outcomes in pulmonary arterial hypertension, potentially necessitating earlier consideration of lung transplantation for carriers of this variant [33]. Further examination of *RNF213* could reveal its involvement not only in cerebral blood vessels, but also in other systemic vasculopathies.

This study has some limitations. First, it included a relatively small sample size and demonstrated potential selection bias, which may limit the generalizability of our findings to a broader population. Further research using a larger and more diverse cohort is necessary to confirm our findings. Second, a primary limitation of this study is the reliance on MRA for the diagnosis of ICAS. Of the 408 cases included in the analysis, only 100 patients (24.5%) were diagnosed using DSA, which is considered a more definitive diagnostic tool. Furthermore, for the subgroup of asymptomatic ICAS, which is the main focus of this article, there were only seven DSA-confirmed cases. Future studies should include a larger proportion of DSA-confirmed cases to enhance diagnostic accuracy and validation. Third, it is important to acknowledge the limitations of this study related to the choice of oral medications, which is ultimately determined by the attending physician. While various lifestyle-related diseases are managed, standard treatments, such as the availability and selection of antithrombotic drugs, have not been established, particularly for asymptomatic ICAS. Moreover, we recognize the importance of considering all relevant factors in the analysis of ICAS, including laboratory variables such as lipids. However, due to the retrospective nature of this study and the inconsistent availability of follow-up laboratory test results in electronic medical records, we were unable to incorporate the laboratory variables. This limitation underscores an area for improvement in future research efforts, and we aspire to conduct further analysis to address this gap.

Advances in genetic testing may enhance the treatment of patients with asymptomatic ICAS. The clinical implications of this study are noteworthy, suggesting that the *RNF213* p.Arg4810Lys variant is a valuable marker for identifying patients with asymptomatic ICAS at increased risk of stenosis progression. This knowledge could assist clinicians in customizing treatment strategies and implementing a closer follow-up for individuals carrying the *RNF213* p.Arg4810Lys variant.

The *RNF213* p.Arg4810Lys variant showed a distinct association with a heightened risk of stenosis progression in asymptomatic ICAS, highlighting a notable clinical disparity. This implied a difference in the vascular stenosis mechanism attributed to the *RNF213* p.Arg4810Lys variant. Additional examination is necessary to explore the functional implications of this variant and its potential application in personalized medicine for patients with ICAS.

## Data Availability

No datasets were generated or analysed during the current study.
